# Fair is fair: We must re-allocate livers for transplant

**DOI:** 10.1186/s12910-017-0186-9

**Published:** 2017-04-05

**Authors:** Brendan Parent, Arthur L. Caplan

**Affiliations:** 1 Applied Bioethics, NYU School of Professional Studies, 7 East 12th Street, Rm 825b, New York, NY 10003 USA; 2 Department of Population Health, Medical Ethics, NYU School of Medicine, 227 East 30th Street, Rm 722, New York, NY 10016 USA

**Keywords:** Liver transplant, Allocation, Fairness, Justice, Median MELD, Transplant ethics, Organ procurement organizations

## Abstract

The 11 original regions for organ allocation in the United States were determined by proximity between hospitals that provided deceased donors and transplant programs. As liver transplants became more successful and demand rose, livers became a scarce resource. A national system has been implemented to prioritize liver allocation according to disease severity, but the system still operates within the original procurement regions, some of which have significantly more deceased donor livers. Although each region prioritizes its sickest patients to be liver transplant recipients, the sickest in less liver-scarce regions get transplants much sooner and are at far lower risk of death than the sickest in more liver-scarce regions. This has resulted in drastic and inequitable regional variation in preventable liver disease related death rate.

A new region districting proposal – an eight district model – has been carefully designed to reduce geographic inequities, but is being fought by many transplant centers that face less scarcity under the current model. The arguments put forth against the new proposal, couched in terms of fairness and safety, will be examined to show that the new system is technologically feasible, will save more lives, and will not worsen socioeconomic disparity. While the new model is likely not perfect, it is a necessary step toward fair allocation.

## Background

As liver transplant was developed in the 1960’s and 70’s, the challenge of organ preservation necessitated that donor livers be located near those in need of a transplant. Groups of local hospitals knew to contact the nearest transplant program when they had a potential liver donor, which set the stage for the locations and reach of present-day organ procurement organizations (OPOs), which are responsible for identifying potential donors, procuring organs from them, and ensuring these organs are delivered efficiently to appropriate recipients [1]. Within these geographically-defined regions, allocation fairness became an increasing concern as both growing demand and the success of liver transplantation caused the demand to outpace supply. Today fairness is still a concern due to a failure to develop an allocation policy for scarce livers that treats them as a national resource.

A nationally agreed upon system was created and refined to prioritize allocation decisions according to disease severity. But today, liver matching remains constrained by the original 11 OPO boundaries. Because some of these regions have greater numbers of deceased donor livers, the system is now creating drastic and inequitable regional variation in preventable liver disease related death rate.

This disparity in access to liver transplants led the Department of Health and Human Services (DHHS), an agency of the United Stated Federal Government, to call for greater geographic liver distribution and reduced inter-transplant program variability. A new region districting proposal – an eight district model – has been carefully designed to reduce geographic inequities, but is being fought by many transplant centers that face less scarcity under the current model. The arguments put forth against the new proposal, couched in terms of fairness and safety, will be examined to show that the new system is technologically feasible, will save more lives, and will not worsen socioeconomic disparity. While the new model is likely not perfect, it is a necessary step toward fair allocation.

## Main text

### The current strategy for distribution and a proposal for an eight district model

Although the existing division of 11 organ sharing regions and 58 donation service areas in the United States are subject to the same recipient priority ranking system – Model for End-stage Liver Disease (MELD) score, which is a validated calculation using objective variables (international normalized ratio, serum creatinine, and serum bilirubin) to predict survival of patients with liver disease [2] – a given patient’s rank currently matters most within the patient’s regional boundaries. Each patient on the waiting list can only receive a liver procured within the region or a liver that other regions do not want. A regional system is necessary because livers have a limited period during which they can be transported, cold ischemic time. A liver obtained in Tampa will never be helpful to a patient in Honolulu, but the existing boundaries reflect more than just limits on liver viability. They are the product of historic relationships between transplant centers and healthcare organizations that come into contact with potential donors [3]. This map was not designed to optimize allocation to patients in greatest need. Because more livers are available in some regions, these regions are able to transplant patients who are less sick, thus leading to fewer deaths in those regions. The difference in estimated risk of three-month mortality without transplant is as high as 60% across regions [3]. Since livers and other organs are donated with the goal of saving American lives and since government policy has long recognized the duty to steward donated cadaver organs as a national resource [4], this risk variation is unjust.

In the late 1990s, DHHS directed the Organ Procurement and Transplantation Network (OPTN) to revise allocation to reflect three objectives, but only the first has received significant attention. The Final Rule states: (1) Set priority rankings…through objective and measurable medical criteria; (2) Distribute organs over as broad a geographic area as feasible; and (3) reduce inter-transplant program variability [5]. In 2002, the MELD score was developed in response to the first objective, allowing physicians to rank patients according to their lab values. Since 2005, patients with a MELD of at least 15 would be listed with priority given to patients with higher MELDs up to the highest numeric score of 40, and then greatest priority given to patients ranked 1A or 1B (above 40). Efforts have been made to broaden sharing *within* each region. However, more than a decade has elapsed with no attention to the requirement to broaden geographic distribution. Some regions are transplanting patients with a median MELD of 23 (including states like Kentucky and Tennessee) and others are transplanting patients with a median MELD higher than 30 (including states like New York and California) [6]. The drastically higher risk of liver-related death for patients in the latter regions has prompted the United Network of Organ Sharing (UNOS) to consider a new proposal designed to distribute livers over as broad a geographic area as feasible and to reduce inter-transplant program variability.

The plan put forth by the UNOS Liver and Intestine Transplant Committee considers the need for better sharing, oversight to prevent abuse, promoting awareness and monitoring impact. To compare the current allocation model to proposed districting changes, the Committee employed linear programming algorithms in the Liver Simulated Allocation Model (LSAM), which is a validated discrete events simulator that estimates outcomes of each system [7]. According to the Committee report, the LSAM uses “historical inputs,” such as “donors and candidates, organ offer acceptance practices, [and waitlist] removals for death or other reasons,” as well as a transport model based on distances between donor hospitals and transplant centers, and between centers and airports, to calculate and compare outcomes between the current regions and proposed changes. The LSAM’s outputs included median MELD at transplant, number and rate of waitlist deaths, total deaths, and percentage of transplants by various demographics [7].

In collaboration with the Scientific Registry of Transplant Recipients -- a federal contractor responsible for providing analytic support to the OPTN -- the Committee analyzed the modeling data and considered different modifications including a four district model, eight district model, and a concentric circles model [8]. With the intention of transitioning from local sharing toward national sharing, the Committee has chosen new districts sensitive to the limits of liver transport time and associated costs that would minimize MELD score disparity across regions. The eight district model performed as well as the four district model while being more cost and time effective, and the concentric circles model was less effective than either of the former [8]. Accordingly, the 11 districts would be transformed into 8, with 150 mile proximity circles to attach areas of higher supply to areas of higher need [3]. The new model would also ensure that a difference of one or two MELD points between a local patient and a distant patient do not result in the costly transport of a liver by granting the local patient additional points for proximity. The eight district model should reduce geographic disparity of MELD at transplant so that no single population bears greater risk, whereas currently, populations bear different risk based on where they live.

Because there is some evidence of MELD inflation through the application of “exception points” in current liver-scarce regions [9], the new plan proposes revisions to exception point criteria to most accurately reflect disease severity. It calls for the establishment of a National Liver Review Board to ensure uniform application of exception points [3]. Furthermore, the proposal requires education and awareness campaigns to ensure understanding of the new system prior to implementation, as well as standardized data recording and analysis to ensure reduction in median MELD disparity and to identify any unintended consequences [3].

The primary impetus for the new proposal is to make liver allocation more fair. Does it succeed? What are the relevant requirements of fairness?

To be fair any allocation system must treat according to medical need without attention to other individual characteristics. The worth of a life is not determined by race, gender, socioeconomic status, ability, or location, and thus these demographics should neither be weighed in allocation nor unduly affected by allocation. Under the current system, location is a strong indicator of whether a person in need of a liver will survive. If the implementation of a new model results in a significant mortality gap between men and women, or between people of different racial identification, or between the wealthy and the poor, then it must be adjusted. Careful attention has been paid by the eight district model’s designers to ensure that geography will not result in such disparities.

Furthermore, the scarcity of a life-saving national resource carries the duty of careful stewardship to ensure the resource is used to provide the greatest benefit. Because there are not enough livers for all persons in need, those who are at greatest risk without a transplant must be given priority. Cold ischemic time makes it necessary to delineate regions in which patients are prioritized against each other according to risk. But there are far more livers in some regions than others. Thus the sickest patients in some regions are much less sick at the time of transplant than are the sickest in others. This means organs are not being allocated to those in greatest need and more people are dying. Geography is not, in itself, a morally relevant variable. Allocation ought to occur according to medical need and without significant benefit disparity among demographic and geographic groups. Reconfiguring organ allocation regions appears to be an effective means for reducing geographic disparity.

Vocal opponents of the new proposal are affiliated with transplant programs in regions with less liver scarcity. There are a few themes that reoccur in their arguments against change: Outcomes would be worse; costs would rise; the MELD data catalyzing the proposal is inaccurate; and the new system would be less fair. The reality of disparity in access indicates that these arguments are likely founded on different motives. It is possible that these motives are the desire to protect the strong financial position afforded to transplant programs in areas with lower liver scarcity, and the more admirable desire to protect one’s own patients who would be forced to wait longer for a liver under the new proposal. With over 17,000 people in need of, approved and waiting for a liver [10], scarcity is a serious problem which necessitates protecting the interests of all Americans over the understandable loyalty physicians feel to their own patients.

### Liver transplant outcomes will improve

Those who claim that the new districts would make outcomes worse point to greater liver travel time and lower survival benefit of transplanting sicker patients [11]. Both points must be considered in context. The proposed eight district model is drawn to respect the limits of liver travel time. Under the new proposal, the number of livers flying would rise but only from 55% to 65%, and they would only travel approximately fifteen more minutes on average. Under the new model, approximately 95% of transplants are projected to occur within the reconfigured districts, with a median transport time of 1.8 h [3]. The generally accepted cold ischemic time for a liver is six to ten h [12], and at least one recent study of 350 liver transplants shows that even cold ischemic time upwards of 12 h did not affect graft or patient survival [13].

The focus on lower survival benefit of sicker transplant patients is equally misguided. It is true that the new districts would raise the median MELD at transplant to between 24 and 29.1 [3], thus cutting regional variance in half, but the survival data for patients at this MELD is not worse than for patients transplanted at the median MELD of regions with the least sick liver transplant patients. In fact, a retrospective analysis of 37 studies including over 53,000 patients concluded that there is a low level of evidence that relative MELD score predicts post-transplant survival [14]. The MELD score was designed to predict risk of death without transplant, and studies have validated that higher MELD indicates a higher waiting list mortality risk [15, 16]. This actually supports the importance of eliminating median transplant MELD regional disparity. Outcome metrics modeled under the eight district model indicate there would be a decrease in the number of overall deaths, including pretransplant, waiting list, and post-transplant, and predict no significant survival differences according to other demographics such as age, sex, or race [3]. Implementing the new proposal will improve, not worsen, liver transplant outcomes.

### Costs will drop

Some costs would likely rise under the new proposal, specifically transportation, and particularly for transplant programs in areas with lower median MELD scores [17]. But this cost is offset by savings in transplant-associated care, and by the value of saving additional lives. Compared with the current allocation system, over five years the eight district model is estimated to increase per-patient transportation costs predominantly due to longer travel time. However, it is also estimated to decrease Medicare spending for waitlisted patients (less time treating encephalopathy, variceal bleeding, infections, and hepatocellular carcinoma), and total cost of actual transplants and post-transplant care because of fewer total transplants [18]. Overall five year costs are expected to drop about 1.3% [18]. Medicare -- which pays for the vast majority of liver transplant related costs -- will be saving money overall, thus taxpayers and Medicare enrollees (including liver transplant patients) can expect that their costs will not rise. Because transportation costs are assigned to a Medicare cost center [18], transplant center reimbursement rates might be adjusted to reflect increased transportation costs without reducing the availability of Medicare resources.

Costs should, of course, be tracked carefully after implementing any new system. But based on current estimates, redistricting to achieve a more ethical allocation system should not impact patients’ financial access to transplants or the capacity for transplant centers that bear additional costs to provide them. If evidence begins to suggest this possibility, then methods for increasing reimbursement rates or creating federal subsidization should be explored.

### Livers are not local

Opponents also say that livers should “stay local” to prevent widening the socioeconomic healthcare gap, and to honor donor intentions [19]. The new eight district model should have no impact on access to healthcare and would better honor expressed donor wishes. It is true that the regions with lowest median MELD at transplant are proximate to populations of low socioeconomic status, and several within those populations likely benefit from their transplant programs. However, programs with higher median MELD at transplant are, despite in some cases being near some of the wealthiest cities in the nation, also proximate to disadvantaged populations in the reconfigured regions. All people in need of liver transplant with access to health care in each region will continue to be prioritized according to their MELD scores. The extent to which the wait-listing process screens people based on ability to pay is a problem regardless of location. There is no data suggesting that discrimination based on financial status is greater in more liver-scarce regions. Furthermore, individuals without access to healthcare, particularly in rural areas, are sometimes not being waitlisted for organs regardless of their region, and while efforts should be made to include these individuals, adopting the eight district model would not affect this population.

Those who claim livers should stay local incorrectly assume livers are already kept local. 47–62.5% of transplant recipients from the five busiest transplant programs in the nation (all among the lowest median MELD at transplant) are not from the program’s community [20]. Patients with adequate financial resources can and do migrate to areas of lower MELD at transplant and often list at multiple centers, which increases the chances of being prioritized [21]. This is not an option for people without the resources to relocate, especially under conditions of poor health. The incentive to relocate would disappear if median MELD at transplant was consistent across regions, thus making allocation more fair across socioeconomic lines.

It might make intuitive sense that potential donors would want their organs to be received by sick members of their own communities, but as just described, the current system does not match this intuition. Donated livers are often not transplanted into local recipients because wealthy recipients relocate to regions with better donor to recipient ratios. Systems like the VA utilize centers of excellence as do some payers, overriding locality. Additionally, a recent survey shows that 82% of respondents would prefer their organs go to the person in greatest medical need regardless of location [22]. Organ allocation policy should not be determined by public preference, but this data indicates that the eight district model would support the autonomous preferences of a significant portion of potential donors. Although livers cannot yet be a truly national resource due to technological constraints of transport time, end-stage liver disease affects the entire population. The eight district model provides more effective treatment nationally than the existing skewed geographic system.

### More donors will not fix allocation

There is regional disparity in the number of registered donors that in some cases corresponds with areas of liver scarcity, but focusing on increasing donor numbers will not fix allocation disparity. New York State has the lowest number of registered donors among the United States [23], and also suffers from one of the highest median MELD scores at liver transplant. Opponents of the new proposal claim that liver scarcity in areas like New York is the consequence of low registered donor numbers, and that it would be unfair to take livers from areas with better registration. Two facts undermine this claim. First, it is an unfortunate truth that many areas with higher procurement rates also have more deaths caused by guns, car accidents, and strokes [24], which are circumstances that provide for donation after brain death. Areas like New York that have better public health infrastructure have less incidents that lend themselves to deceased liver donation, regardless of the number of registered donors. Although beyond the scope of this paper, it would be best if the geographic disparity in liver availability were reduced by implementation of additional public health measures thus leading to fewer deaths overall. When this occurs, we will still be morally obligated to ensure that liver distribution prioritizes those in greatest need.

Second, even if OPOs got every eligible person to donate, there would only be an additional 1,300 livers available over a six year period [25], which hardly makes a dent in the context of over 17,000 people on the waiting list [10]. However, there is no question that efforts must be made to increase donation rates, because those 1,300 additional livers nationally might save 1,300 additional lives over six years.

There is a valid argument based on reciprocity that a community desiring access to organs for transplant should demonstrate commitment to donation. But the problem with using the reciprocity argument to restrict a state’s access to transplant on the basis of low registered donor percentage is that a low registration rate is likely not perfectly correlated to the number of willing donors. There is some evidence to suggest that there are more people willing to be donors than there are registered donors, for reasons including not knowing how to register, or not thinking of oneself as healthy enough to donate [26, 27]. This suggests there are other reasons for low state donor registration, which might include ineffective education campaigns and/or provision of inadequate registration opportunities. In light of this, it is not fair to punish sick New York residents -- almost 4 million of whom are registered donors [23], and potentially more who might be willing to donate but do not have the necessary education or opportunity to register -- for their state’s inability to convert willing individuals into registered donors.

### The median MELD and exception points

There is evidence to suggest that patients are receiving inflated MELD scores through the assignment of “exception points,” which are supposed to be awarded in cases when MELD factors are not sufficient to represent the patient’s risk of pretransplant mortality [9]. But there is no evidence to suggest that the practice is isolated to regions of greater liver-scarcity. The most prominent example is additional points awarded to patients with Hepatocellular Carcinoma (HCC), which is the most common form of liver cancer. The logic goes that because conditions like HCC are not associated with worsening liver function but do indicate higher risk of death without transplant, affected patients should receive exception points to better compete with other patients who have more obviously failing livers [28]. Despite intentions to improve fairness, the result has been a steady increase in median MELD across regions and comparatively superior outcomes for patients with HCC-related exceptions across regions. Contrary to the claims that this practice is more common in more liver-scarce regions [11], a study of close to 79,000 liver transplant patients between 2005 and 2012 shows that regions with the *least* scarcity have the greatest percentages of waitlisted patients with exception points [28]. Furthermore, vastly greater numbers of waitlisted patients in the most liver-scarce regions die each year. So while the practice of awarding exception points might need to be reexamined, current practice is not unfairly benefiting patients in any specific regions. The new proposal would create a National Liver Review Board to ensure consistency in the exception point award process across regions. The UNOS Liver and Intestine Committee has submitted new exception point guidance that the National Liver Review Board would use to assess exception requests [3]. Independent of the primary impetus to reconfigure districts, the establishment of a national Board is an important move toward justice.

## Conclusion

Examining what different stakeholders have to gain or lose by the implementation of the eight district model reveals the understandable (but easily overcome) motivations behind the opposition. Some larger centers will see less revenue. Some smaller centers serving more remote populations might not be able to maintain their transplant programs [5]. Logistical complexity will increase because more organs (and potentially surgical teams) would require transportation for longer distances. OPOs will need to re-coordinate their operations. The hurdles, however, are but minor necessities in light of the expected benefits. Existing regions of comparatively low donor to recipient ratios will have access to more livers for their comparatively sicker prioritized patients. Nationally, the redistricting proposal will significantly reduce the variation in median MELD at transplant, reduce the prevalence of the sickest candidates thus reducing suffering, and is expected to save over 300 lives per year [5].

Transplant programs in less liver-scarce regions do not want to incur the additional expense, loss of revenue, and complications that the eight district model would entail. It is possible that reimbursement rates could be altered to capture the revenue loss as livers are moved to areas of greater need. But what price are we not willing to pay, and what revenue should we not be willing to sacrifice, to reduce suffering and save hundreds of lives? We might not be able to expect individual physicians to adopt a public health or national stewardship mindset; we probably do not *want* them to do so as good advocates for their patients. However, justice requires that under conditions of a widespread need, and severely limited resources involving a national resource, the preferences of individuals with lower MELD scores to be transplanted sooner cannot supersede the obligation to save more total lives. It is the duty of government, in this case the OPTN, to capitalize on the technical capacity to improve liver allocation fairness at a national level, and to monitor the impact of change. The new eight district model might not be the best allocation system, but it is a step toward fairness.

## Abbreviations

DHHS: Department of Health and Human Services
HCC: Hepatocellular Carcinoma
MELD: Model End-stage Liver Disease
OPO: Organ Procurement Organization
OPTN: Organ Procurement and Transplantation Network
UNOS: United Network of Organ Sharing
